# Paleopathology of Human Tuberculosis and the Potential Role of Climate

**DOI:** 10.1155/2009/437187

**Published:** 2009-04-05

**Authors:** Andreas G. Nerlich, Sandra Lösch

**Affiliations:** Division of Paleopathology, Institute of Pathology, Academic Hospital Munich-Bogenhausen, Englschalkinger Str. 77, 81925 Munich, Germany

## Abstract

Both origin and evolution of tuberculosis and its pathogens (*Mycobacterium tuberculosis* complex) are not fully understood. The paleopathological investigation of human remains offers a unique insight into the molecular evolution and spread including correlative data of the environment. The molecular analysis of material from Egypt (3000–500 BC), Sudan (200–600 AD), Hungary (600–1700 AD), Latvia (1200–1600 AD), and South Germany (1400–1800 AD) urprisingly revealed constantly high frequencies of tuberculosis in all different time periods excluding significant environmental influence on tuberculosis spread. The typing of various mycobacteria strains provides evidence for ancestral *M. tuberculosis* strains in Pre- to early Egyptian dynastic material (3500–2650 BC), while typical *M. africanum* signatures were detected in a Middle Kingdom tomb (2050–1650 BC). Samples from the New Kingdom to Late Period (1500–500 BC) indicated modern *M. tuberculosis* strains. No evidence was seen for *M. bovis* in Egyptian material while *M. bovis* signatures were first identified in Siberian biomaterial dating 2000 years before present. These results contraindicates the theory that *M. tuberculosis* evolved from *M. bovis* during early domestication in the region of the “Fertile Crescent,” but supports the scenario that *M. tuberculosis* probably derived from an ancestral progenitor strain. The environmental influence of this evolutionary scenario deserves continuing intense evaluation.

## 1. Introduction

Today, tuberculosis is still a major predator of mankind causing every year millions of deaths worldwide. Despite the enormous social and economic burdens, the origin as well as the evolution of the pathogen is still far from being completely understood. This includes the understanding of the mechanisms of virulence, the host-pathogen interaction, and the bacterial development, including the influence of changing environmental conditions, including climate. 

Tuberculosis as a chronic infectious disease is caused by mycobacteria. These mycobacteria are able to induce a chronic destructive inflammation that typically contains granulomas with central necrosis (the “tubercle”). Due to local and/or systemic spread principally every organ can be affected. Frequently, any systemic spread by the blood stream involves bone tissue with the most preferred osseous lesions occurring in the vertebral bodies. 

Out of hundreds of mycobacterial strains—that mostly exist as soil bacteria—only four strains are the main infectious agents for human tuberculosis. All four strains, termed *M. tuberculosis, M. bovis, M. africanum, M. canetti*, lead to an identical clinical pattern. Few other mycobacteria, such as *M. avium* and *M. kansasii*, may also induce granulomatous pulmonary infection, frequently, but not exclusively in immunocompromised patients, such as in HIV-infected individuals. These mycobacteria are counted into the atypical mycobacterioses.

Until recently, the aspects of origin and evolution of pathogens were restricted to the comparative analysis of present isolates and the extrapolation of the resulting observations to evolution and origin. Despite considerable efforts, the resulting data remain hypothetical. The most recent technical improvements of polychain reaction (PCR) techniques, however, now allow the identification and characterization of gene segments in biomedical remains of hundreds to thousands of years of age [1]. This has been made possible since PCR can amplify even minute amounts of DNA, such as the very small amounts of intact DNA-molecules residing in ancient biomaterial, for example, in bone or mummified soft tissues. This novel approach offers not only potential insight into the evolutionary time course of distinct strains of mycobacteria, but possibly provides also correlative data on environmental influences on mycobacterial development and its diversity. 

In this report we describe the current status of ancient mycobacterial DNA-analysis in ancient human remains with particular reference to the present scenario of the evolution of human tuberculosis and propose initial assumptions on the influence of environment factors.

## 2. Previous Theories of the Evolution of Human Pathogenic Mycobacteria

Human tuberculosis has previously been assumed to have come from the close contact between humans and animals, especially from either bovine or caprine source. Accordingly, the bovine form of mycobacteria (*M. bovis*) had previously been suggested to be the initial evolutionary form of mycobacteria [2]. In consequence, the climatic changes in the Near East/North Africa with desert formation in distinct areas and the formation of the so-called “Fertile Crescent” lead to the gathering of nomadic populations and the formation of sedentary settlements. Along with this change in human live style, animal domestication took place. This was regarded to be an initializing event of TB transmission to humans. In consequence it was believed that the bovine form is “older” and thereby potentially less virulent to humans. This, however, is not consistent with current clinical observations. 

**Figure 1:**
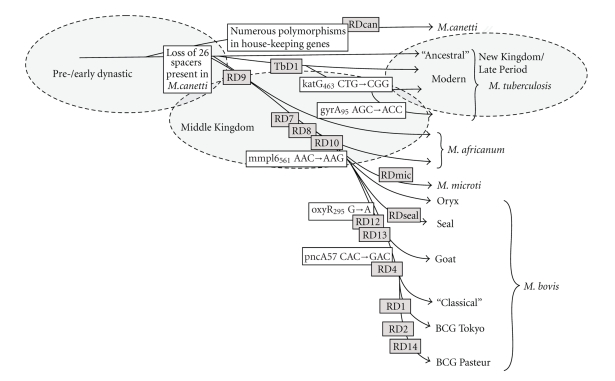
*Schematic diagram of the potential evolution of the various tuberculosis strains (modified according to Brosch et al. [3]) and its impact on ancient Egyptian findings*. The circles indicate the presumed location of the TB main strains as identified by spoligotyping and typical mutations in the various ancient Egyptian populations.

First doubt whether this hypothesis is correct or not came from a recent comparative genomic analysis of various strains of pathogenic mycobacteria. Accordingly, Brosch et al. [3] compared distinct gene segments of currently available mycobacteria of the MTB-complex and suggested that the presence/absence of these segments were due to evolutionary development. The comparative analysis hypothesized that a human strain of mycobacteria, *M. tuberculosis*, represents the “most ancient” strain. During the later development the other pathogenic mycobacteria split off including the bovine type (Figure 1). This intriguing hypothesis, however, can provide neither any time frame nor any spatial distribution of the different developmental stages in the history of mankind. As well no data can be deduced to indicate any potential environmental influence on mycobacterial evolution.

Accordingly, only the direct molecular analysis of ancient human (and animal) biomaterial is able to solve these questions. Furthermore, such a molecular analysis may also answer two other major questions: (i) was human tuberculosis a rare or a frequent disease in ancient populations? and (ii) which strain(s) occurred at which time period in ancient populations? The answers may also provide a basis for understanding the influence of any environmental factor on mycobacterial evolution.

## 3. The Pathomorphology of Osseous Tuberculosis

In its very typical form osseous tuberculosis is a chronic destructive inflammatory process with tubercle formation in bone comparable to affection of other organs/tissues. The predilection sites of osseous tuberculosis are not only the (thoracic and lumbar) spine and the epiphyses of large joints, but also the skull (tuberculose meningitis) and various small and flat bones. In general, every bone may be affected. The spinal affection seems to result from a spread from the thorax (pulmonary and pleuritis tuberculosa) to the adjacent vertebral bodies (via lymphatics and the paravertebral venous plexus) or the systemic spread by the blood stream. The latter may explain the higher frequency of tuberculose arthritis and the involvement of the epiphyses. 

**Figure 2:**
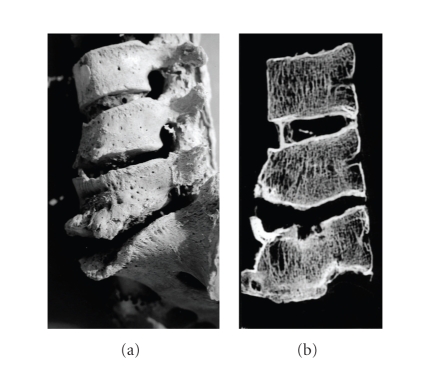
Typical macrophology of spinal tuberculosis with (a) ventral destruction of the affected vertebral bodies and (b) fistular defects of the bone.

The end stage of tuberculosis of the spine is the very typical ventral collapse of the vertebral body leading to a more or less severe angulation of the vertebral column (“gibbus”) (Figure 2). This condition indicates long-standing bone tuberculosis; the lesions are typically characterized by severe alteration of the ventral side of the vertebral body and the presence of fistulas of the bone and/or the intervertebral disc. Major differential diagnoses comprise trauma sequels and some other spinal infections, such as brucellosis. While trauma-induced defects mostly affect the ventral and dorsal sides of the vertebral body, brucellosis does not show fistular defects.

**Figure 3:**
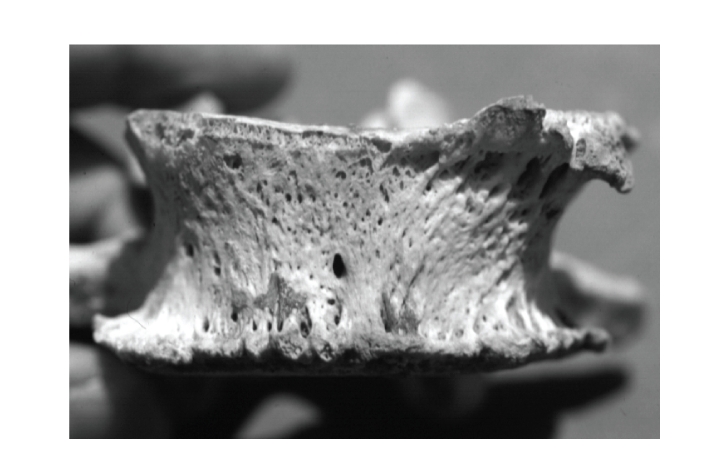
Macromorphology of nonsepcific pathological alteration of the ventral side of the vertebral body, which is still suggestive for a very early phase of osseous tuberculosis.

Recently, we [4] have identified a considerable number of less typical osseous lesions, particularly of the spine, which proved molecularly positive for the MTB-complex. These lesions are frequently characterized by an irregular pitting of the ventral vertebral body, but lack of vertebral collapse and formation of fistulae (Figure 3). The differential diagnosis of those lesions includes trauma sequels and a variety of different infectious diseases, including mycobacterioses. Previous molecular studies on those “nonspecific” vertebral suggest that mycobacterial infection may represent a major part of those lesions [4, 5].

## 4. First Evidence for Tuberculosis in Human History

The presence of tuberculous infections in human history was first evidenced by very typical macroscopic changes of infected bones, mainly of spinal lesions (see Section 3), which have been found since the Neolithic period (i.e., approx. 3000–7000 BC). One of the earliest observations of typical spinal tuberculosis in Europe comes from the cave of Arena Candide (Liguria) in Italy where the material was dated to the first half of the fourth millennium BC [6]. Slightly “younger” are cases of comparable morphology from Denmark [7] and Germany [8]. Very recently, a combined morphological and molecular investigation on a Neolithic finding from the Mediterranean costal region of Israel described two individuals, mother and child, presenting with suspicious bone pathology for TB which was confirmed by molecular analysis (see Section 8). These two cases date back to approximately 7000 BC [9]. Significantly more cases have been identified on the basis of the spinal morphology in the Roman Period and the Middle Ages. Currently, it is believed that the number of tuberculosis infections here generally increased in the period following the growth of the population—especially in the expanding townships—following the hunter and gatherer populations of the Neolithic period. However, there exists as yet no proof for that assumption. Most of these observations are based merely on morphological analysis, but still await molecular confirmation. Furthermore, some data base has been gathered from skeletal analysis of some populations from Middle Europe (Germany, Hungary, Latvia, France) from the time periods between Middle Ages and recent centuries.

In contrast to Europe, much more information is available for those regions where accidental or intended mummification occurred, especially in South America and Egypt. Here again, typical morphological lesions of bone and/or residual soft tissue indicated the presence of human tuberculosis since early periods (c. 3500 BC). While only few cases of the Pre-Columbian mummies have as yet been analyzed by molecular techniques providing a successful amplification of tuberculosis ancient DNA [10–13], much more data are available for the Egyptian material [5, 14–18]. Therefore, it makes sense to have a closer look at the current data-base in the Egyptian material. Unfortunately, as yet no successful molecular investigation has dealt with populations having a hunter-gatherer lifestyle, predating animal domestication and agriculture. The “oldest” case of molecularly proven infection with mycobacteria goes back to a c. 17,000 years old bison [19], but no human infection that is older than 9,000 years before present has been successfully analyzed.

## 5. Evidence for Human Tuberculosis in Ancient Egypt

The first evidence that human tuberculosis was present in ancient Egypt came also from typical macroscopic osseous changes in human remains, which were in this instance the well-preserved Egyptian mummies. Likewise, one of the first “cases” has already been described in 1910 [20]. The mummy of the Amun priest Nesperhan presented with typical ventral destruction of the lower thoracic spine leading to the typical gibbus formation of spinal tuberculosis (see above). Additionally, ossified paraspinal abscesses along the paravertebral muscles were present in this mummy. Besides this very typical case some other samples of spinal lesions were detected. In a previous compilation, a British group [21] was able to summarize 31 cases of presumed spinal tuberculosis in published material from ancient Egypt.

In 1997 our study group was the first that identified ancient DNA fragments specific for the human *M. tuberculosis* complex in a mummy with typical morphological signs for spinal tuberculosis [14]. Due to this case, we were able to improve the analytical technology allowing to analyze the molecular characteristics of ancient human tuberculosis.

## 6. The Molecular Analysis of Human Tuberculosis—Technical Prerequisites and Avoidance of Contamination

The analysis of ancient DNA (aDNA) is always risky with respect to contamination with recent DNA, since the first is fragmented and mostly very poorly preserved, while the latter is well preserved and therefore much easier amplified/analyzed. Therefore, even very minor amounts of recent DNA can produce artefacts. In order to minimize the risk of contamination a series of conditions have been claimed and all studies from our own laboratory—as well as other published material—have to meet those criteria. These include: (i) whenever possible, removal of samples on-site; (ii) storage of samples in sterile lab ware; (iii) all lab work only in rooms that are exclusively dedicated to aDNA analysis; (iv) removal of the sample surface (by chemical and/or mechanical treatment); (v) wherever possible, use of single-use lab ware; (vi) sterile bench conditions and extensive precautions for the lab personal to avoid carry over of material; (vii) replication of data in a second, locally distant laboratory. 

The criteria for the work with human aDNA have to be kept extraordinarily strict, which seems to be less at risk for the work with mycobacterial aDNA since the modern day contamination sources are restricted to laboratory carry-over effects (therefore, use of rooms dedicated only to aDNA analysis), but less to persons that might be infected with the bacteria. In all our own studies—that are part of the present review—those criteria have been strictly obeyed. Furthermore, an additional “control” for the mycobacterial analysis is presented by the MTB-strain analysis (e.g., by spoligotyping) which might have rapidly detected contamination with specific (recent) strains. Similarly, a carry-over contamination of the pre-PCR material with post-PCR fragments is (at least in those studies performed by our own group) not very likely since in that case identical spoligotyping patterns of several cases should have been expected. This was not the case in our studies. (In other studies, such as that by Hershkovitz et al. [9], the spoligotyping provided only incomplete and not sufficiently conclusive patterns which may be due to less optimal preservation of the material than in the “younger”—and artificially mummified—Egyptian biomaterial). Although we have accordingly no evidence that the contamination problem affected most data shown in this compilation, it cannot be fully ruled out that ancient contaminations occurred that might have influenced any observation.

## 7. Molecular Estimation of Tuberculosis Frequency in Various Ancient Egyptian Populations

Meanwhile our study group has analyzed 160 samples—mostly bone samples of the spine—for the molecular presence of tuberculosis. This material came from two sites, the pre- to early dynastic necropolis of Abydos, Upper Egypt (c. 3200–2800 BC), and several tombs of the “Necropolis of the Nobles” of Thebes-West which cover a time period from the so-called “Middle Kingdom” (2000–1600 BC) and the “New Kingdom” (1600–1000 BC) until the “Late Period” (800–500 BC). Out of this material three major time periods could be evaluated separately: the pre- until early dynastic period (*n* = 13), a tomb exclusively used in the Middle Kingdom period (*n* = 45) and several tombs built in the New Kingdom and used until the Late Period (*n* = 102). In this material we detected in 4 of the 13 pre- to early dynastic cases analyzed, in 13 of 45 samples of the Middle Kingdom, and in 16 of 102 New Kingdom until Late Period samples specific mycobacterial ancient DNA of the tuberculosis complex (Table 1). 

Although there are differences in the preservation status of the biomaterial between the predynastic period until the Late Period (mainly due to advancing techniques of mummification/conservation), these studies reveal very similar tuberculosis frequencies of approximately 30% (pre-/early dynastic), 29% (Middle Kingdom), and approximately 20% (New Kingdom until Late Period) (differences statistically not significant) suggesting comparable infection rates in the various populations of ancient Egypt over a time period of almost 2500 years. 

It has, however, to be taken into consideration that (i) the overall numbers of samples of these studies are still very low so that any generalization must be made with great care and (ii) we initially selected samples for their morphological appearance suggestive for osseous tuberculosis. Thereby, at least at the first glance a certain selection bias was introduced into our analysis which, however, was not different between the various subpopulations so that those data may be comparable between each others, although only to limited extent to other molecular studies. Accordingly, we also analyzed the results of tuberculosis frequencies with respect to the morphological appearance of osseous lesions. In more than 75% of those cases with the very typical morphological features of tuberculosis (e.g., typical spinal “gibbus”, Figure 2) the molecular analysis provided a positive result. Even in those cases with slight, but nonspecific osseous lesions (see above, Figure 3) even 20% of cases were tested positive, so that an overall estimate of approximately 40% of typical/suspicious cases was positive for TB aDNA (Table 1). Most surprisingly, almost 10% of bone samples with unremarkable morphology provided a positive molecular result. The latter result is explained by the fact that pulmonary tuberculosis may lead to a systemic spread with the blood stream even without forming typical morphological lesions, for example, in the ultimate premortal time period. This has been confirmed by a recent own study including a series of recent autopsy cases with autoptical evidence for “systemic” spread (in this case: lymph node affection beyond the most intimate lymph node of the primary complex, miliar tuberculosis, or Landouzy sepsis) showing a high percentage of molecularly positive results in otherwise morphologically unremarkable vertebral bodies. In consequence, the molecular identification of *M. tuberculosis* complex aDNA suggests a premortal systemic spread of the bacteria.

In addition, those various “study populations” represent interesting models to calculate the rough infection rates in ancient populations, such as those from ancient Egypt. Supposing a comparable frequency of macromorphologically detectable affection of bone by systemically spreading TB (in present day populations approx. 5%, McTammany et al. [25]), the macroscopic identification of 12 typical cases in c. 1.000 mummies and skeletons (predynastic until Late Period) indicates a tuberculosis prevalence of approximately 25% of the populations. Unfortunately, the distribution of “typical TB cases” is not even in the different time periods analyzed and some populations even do not show those typical cases so that differences in the TB infection rates cannot be deduced until now.

## 8. Molecular Analysis of Tuberculosis in Other Historic Populations

Meanwhile, several laboratories have successfully identified tuberculosis DNA in a broad range of populations from differing time periods, for example, Neolithic Israel [9], Pre-Columbian Peru [10] and Northern Chile [11–13], Byzantine Turkey, Pre-European contact Borneo and Romano-British England [26], Medieval England [27, 28], Medieval Lithuania [29], and 18th-19th century Hungary [30]. Our study group contributed studies on Medieval Hungary [4, 23], Southern Germany [17, 24], and Southern France [22].

Out of these studies only few reports cover a series of cases, most studies cover still case reports or summarize few isolated cases without providing a population basis. Beyond this, the analysis of a Lithuanian series [29] describes tuberculosis infection on a population basis with approximately 25% tuberculosis prevalence which is very comparable to a series of natural mummies from Christian Nubia [31]. Similarly, observations on a Middle Age population from Southern Hungary (Avar period, c. 700–900 AD, Bacsalmas, Hungary) have been done on a total of 46 out of 480 cases [23]. Again, we detected a TB frequency with approximately 30% which is in the range of the Egyptian material (Table 1). 

A recent molecular study on several Hungarian natural mummies (AD 1731–1838) revealed in 55% of that population (*n* = 168 individuals investigated) a positive result for tuberculosis [30, 32]. There was a greater proportion of tuberculosis positive cases in mature adults than in senile and adolescent ones. Due to the excellent conservation the successful amplification of ancient DNA in this population was exceptionally well.

Further, but mostly small studies on samples from Southern France (La Celle, Provence [22] and Southern Germany (Rain/Lech; [17], and Sulzbürg [24] reveal comparable infection rates as in the other series suggesting the presence of mycobacterial DNA in human populations dating back to approximately 3200 BC at a surprisingly high prevalence level for tuberculosis infections with comparably high numbers. 

In summary, all studies using the molecular analysis of identification of *M. tuberculosis* in historic populations between 3.200 BC and 1800 AD suggest constantly high infection rates with an increase only in the last few centuries (e.g., Hungary, which might be significantly influenced by the excellent preservation of the Hungarian mummies). In terms of influence of environmental factors, therefore, the significant differences like, for example, in climate, nourishment, or social stratification obviously does not influence the infection rates of this disease. However, since all populations investigated as yet belong to populations in settlements, this may be responsible for the high prevalence of TB.

## 9. Molecular Identification of Tuberculosis Strains in Ancient Egyptian Material

Besides the molecular identification of presence or absence of human-pathogenic mycobacterial DNA, the analysis of the tuberculosis strains is of utmost interest. This especially is necessary to evaluate evolutionary aspects of the mycobacteria and to identify the spatial and temporal distribution of the infections in various human populations. At present data on the mycobacterial strain distribution are available for ancient Egypt [15, 16], prehistoric Siberia [33], Medieval Britain [34], and the Hungarian mummy population (see above [30, 32]). The data were obtained either by the analysis of strain-specific gene segments of the human pathogenic mycobacterial strains (e.g., genes that occur only in one strain or genes that show specific gene variance at specific spots) or by a special amplification procedure where the presence/absence of spacer regions of the IS6110-gene is determined. The latter procedure, termed “spoligotyping”, provides a typical signature of spacers that is unique to different mycobacteria strains. It is of note that both approaches show less high numbers of successful amplifications as compared to the usually investigated multicopy IS6110-gene (the marker for presence/absence of tuberculosis DNA) since the “drop-out” of gene regions affects these regions more easily than that of the multicopy gene IS6110 in total. Therefore, not every case with successful IS6110-amplification also shows a positive mycobacterial strain identification.

In the “oldest” material from ancient Egypt [18], only few successful spoligotyping results could be obtained from the pre- until early dynastic material (Abydos, c. 3200–2800 BC). Accordingly, we were able to obtain 2 complete and 4 incomplete spoligotyping patterns in the pre-/early dynastic material. However, more data are available for the Middle Kingdom (c. 2000–1600 BC) and the New Kingdom until Late Period (c. 1500–500 BC). The resulting data showed a significant variance of strains with those that are at present widespread over the whole world and those that are restricted to specific areas. Interestingly, in none of the as yet successfully amplified 160 cases *M. bovis* was present. To this respect, one has to take into consideration that *M. bovis* may have escaped much more easily the molecular analysis than *M. tuberculosis*, since *M. bovis* contains fewer copies of the IS6110 sequence than *M. tuberculosis*. Furthermore, the pre- to early dynastic material revealed an “old” strain of *M. tuberculosis (typus humanus)*, no mutations in den katG and gyrA genes and no deletions of TbD1 and RD9; *n* = 2 [18], which is slightly different from the widespread “modern” *M. tuberculosis* strain in spoligotyping (mutations and deletions present), the Middle Kingdom material additionally *M. africanum* strains, and the New Kingdom until Late Period material a “modern” strain of *M. tuberculosis (typus humanus)*, but not the “old” strain seen in the pre- to early dynastic period. Since these observations are coming from different tombs (and two different burial sites) we cannot conclude a direct evolutionary line. However, the data are very well in agreement with the hypothetical assumptions by the Institute Pasteur group [3, Figure 1] suggesting that a “switch” between “old” to “modern” *M. tuberculosis (typus humanus)* occurred between c. 2000 and 1500 BC. *M. africanum* is also seen in a very early population so that its role in early evolutionary processes is plausible (possibly representing an evolutionary parallel development to the *M. tuberculosis*). Finally, it is of highest interest that *M. bovis* is (at least until now) not seen in any ancient Egyptian sample analyzed up to now.

Interestingly, the as-yet oldest molecularly proven human case of mycobacterial infection [9] showed at least some features of a “modern” pattern of mycobacteria. Thus, the authors were able to identify a TbD1 deletion. In consequence, the aforementioned “evolutionary scheme” may be subjected to differences in the time scales of various regions and/or populations. At present, the number of molecularly investigated cases is too small to allow final conclusion and eventual surprises are not excluded.

However, well in line with all the observations, the Medieval British and the Hungarian mummy populations revealed only *M. tuberculosis (typus humanus)* strains, but not *M. bovis*. Here, no *M. africanum* was detectable. However, very recently, Taylor et al. [33] described four cases of *M. bovis* infection in skeleton from a Siberian Iron age cemetery (dating back c. 2000 years BP) suggesting that this strain originated in the East with more recent spread to the West. To this respect, it has to be investigated not only whether special features of environment, such as distinct living conditions, contact between animals and humans, but also whether climate may have been influential factors for the development of the *M. bovis* strain and the subsequent infection of humans.

## 10. Possible Evolutionary Scenario from Theoretical and Paleomicrobiological Analyses

The growing puzzle of data offers new insight into the distribution and development of human pathogenic mycobacteria. We thereby suggest that an “old” strain of the human type of tubercle bacteria was (at least one of) the first original strain. It is still plausible that this was acquired from animal source, be it of caprine or bovine origin. This question, however, remains open until adequate animal material is investigated. There exists at least one hint for this hypothesis: in a case study Rothschild et al. [19] reported *M. tuberculosis* DNA in bones from a 17,000 years old bison. The genotyping of this observation furthermore suggests a genotype which is closer to *M. tuberculosis* (human type) than to any other mycobacteria. In consequence, the analysis of animal residues originating from the period of early domestication is of particular interest to substantiate this issue.

In addition, our identification of *M. africanum* in a tomb complex that had exclusively been used in the Middle Kingdom, as well as in a further Middle-New Kingdom tomb from Thebes-West, is also of particular note since this type of mycobacteria is assumed to originate from Central/Eastern Africa and a transmission to Egypt in the indicated time period of intense trading connections to the Sudan area is plausible.

Our data of the New Kingdom until Late Period material shows a dominance of “modern” type of *M. tuberculosis* strains, such as those that dominate the present day isolates affecting humans. Accordingly, we have good evidence that the “transition” from “old” to “modern” *M. tuberculosis*—already suggested by Brosch et al. [3] on a hypothetical basis—indeed happened and that this may have occurred between the early dynastic to New Kingdom period, that is, in a time frame of about 1500 years. Further analysis may narrow this frame even more (see also Figure 1).

## 11. The Climate in the Historic Nile Valley

Without the river Nile, Egypt would be (and have been) a complete desert region (except for the coastal areas). The unique geographical condition of a several thousand kilometres long “river oasis” was fundamental for the origin of the ancient Egyptian empire and its persistence for several millennia. Further to the mere transport of water into a completely arid region (with less than 5 mm precipitation/m^2^ per year in the South) the river Nile provided the country with an extremely fertile soil. Since the yearly Nile flood was so essential for the ancient Egyptians, the level of the river was precisely recorded. Dating back to the year 622 AD we have fairly precise measures of Nile water gauges, further, though incomplete information goes back to c. 3.000 BC. This is an important source of information for the paleoclimate of the region. Further data come from comparative investigations in Europe (e.g., glaciological analyses of Alpine glaciers) and the stable isotope spectra obtained from human remains from various time periods of ancient Egypt. Although these datasets result from very different approaches, they provide us with a rather rough, but interesting climatic pattern in pharaonic Egypt.

Especially the observations of the so-called “nilometer” at the island of Elephantine (Assuan) at the southern entry point into ancient Egypt are of significant use. Likewise Hassan [35] and Riehl and Meitín [36] provide us with a precise change in the Nile floods between c. 620 AD and 1950 AD showing short-period climate changes of the Nile River. These studies clearly showed 8 events of climate variation with duration of ca. 100 years each which was temporarily interrupted during the “little climatic optimum” between c. 1300 AD and 1600 AD. Similar changes have been deduced from the record of the Palermo Stone that indicates similar climatic changes going back to 3000 BC [37]. These data—together with sediment analysis of the Lake Qarun in the Fayum depression which is fed by annual overflows of the Nile—indicate a significant climate change in predynastic ancient Egypt at c. 4200 BC, 3800 BC, and 3000 BC. Resulting prolonged aridity was also determined by approximatly 1 m lower annual Nile floods [35] causing persistent drought in Egypt. This must have significantly influenced daily life in ancient Egypt.

The second set of evidence comes from glaciological studies in the Alpine region where the investigations provide evidence for a cooler and relatively rainy period in the Near Eastern (Ugarit, Syria) [38] along with sediment changes in the Euphrates River at that period. Although these data do not directly represent the Nile region, it seems fair to assume that the climate change during that period may have also influenced the Northern part of Egypt as well (Lower Egypt). 

Finally, the stable isotope pattern of human remains from predynastic (c. 3200–3000 BC) to First Intermediate Period (i.e., c. 2200 BC) and Late Period to Roman findings (i.e., c. 500–30 BC) also suggests climatic changes in ancient Egypt [39]. These investigations used the pattern of stable oxygen isotopes to find out that the paleoclimate in the predynastic period was moister than in the period between the Old Kingdom and the Middle Kingdom (the so-called First Intermediate Period, c. 2200–2100 BC) and also the Late until Roman Period. The findings suggest that there was a temperature decrease from predynastic to First Intermediate Period and a temperature increase until the Late to Roman Period. 

Taking these various pieces of (mostly indirect) evidence for the paleoclimate of ancient Egypt together we can identify cyclic minor climate changes along with a more significant worsening of the climate very early and an amelioration of the climatic conditions later. 

Due to its central significance, the environmental conditions are essential factors for the evolution of all organisms. This holds particularly true for bacteria that live under a severe selection pressure in terms of host-pathogen interaction and virulence. To this respect the climate, that is, particularly environmental temperature, humidity, and so forth, plays a central role. Although this role and its interaction have not been systematically investigated our very preliminary data (on a very restricted study population) provide us with some beginning insight into the interaction of climate conditions and pathogens. With the advent of molecular techniques and the direct analysis of the traits of pathogenic bacteria in human remains from historic populations, we have now the opportunity to broaden our insight into the interaction of “climate” and bacterial evolution. Due to the technical requirements up to now only very few pathogenic bacteria and small series of samples have been analyzed with a central focus on mycobacteria, that is, human tuberculosis. Nevertheless, this unique approach offers us first interesting observations.

- Several studies on the human remains from various time periods and regions (mostly in Northern Africa and Central Europe) suggest constantly high prevalence rates in the study populations.
- Due to the significantly different environmental conditions both of the host and the pathogen (e.g., in terms of “climate”), this suggests that the virulence of the mycobacteria was not significantly influenced by any environmental change—at least since the living habitats of ancient populations were not considerably different. This, however, also means that changes in the living mode, for example, changes from hunter-gatherer cultures to township populations, may be a much more central issue with diseases development and bacterial evolution.
- There are some very interesting aspects of strain development of the *M. tuberculosis* complex which are linked to distinct time periods and regions. A closer analysis of the concomitant environmental factors will be an important issue to identify modulatory conditions.

In summary, although we are at the very beginning of understanding the evolution of bacteria and their interactions between hosts and environment, the paleopathological approach seems to be a highly interesting one also in terms of finding out what conditions have positive or negative influence on distinct evolutionary pathways. Hopefully, the advances in paleopathological techniques will enable us to answer these questions.

## 12. Perspectives

Recent advances in the molecular detection and characterization of microbes in ancient tissue samples provide a new insight into the distribution and evolution of distinct pathogens during human history. Thereby, the direct paleomicrobiological analysis of human remains provides a more and more precise picture of human tuberculosis and the underlying mycobacterial strains. Preliminary studies confirm that modern PCR techniques are capable directly analyzing this issue. Furthermore, it is becoming clear that recent theoretical models of mycobacterial evolution—based on strain differences of recent isolates—indeed occurred in history. Although the picture provides still some isolated and not well linked pieces of puzzle, we suggest an origin of *M. tuberculosis* strains (“old” strains) in the dawn of civilization, but not that of *M. bovis* as previously assumed. Possibly originating from “old” strains, some modern types of *M. tuberculosis* evolved—with some differences in the local and spatial distribution over time and various regions. Ongoing and future paleopathological and paleomicrobiological investigations will hopefully contribute to this picture. 

## Figures and Tables

**Table 1:** Molecular paleoepidemiology of tuberculosis in various historic populations.

Localization (country)	Period/dating	Morpho-logically typical cases* (*n*)	Morpho-logically suspective cases* (*n*)	Molecu-larly analyzed cases (*n*)	TB-pos/typical + suspective cases (*n*)	TB-pos./insuspective cases
Abydos (Egypt)^A,B,D^	3000–2500 BC	2	6/189	13	2/8 (25.0%)	2/5 (40.0%)
Thebes (Egypt)^B,D^	2000–1600 BC	1	12/211	45	12/13 (92.3%)	1/32 (3.1%)
Thebes (Egypt)^A,D^	2000–500 BC	3	30/226	56	3/33 (9.1%)	0/23 (0%)
Thebes (Egypt)^A,D^	1600–500 BC	5	18/519	46	13/23 (56.5%)	5/23 (21.7%)
La Celle (France)^E^	500–1200 AD	2	9/105	11	5/11 (45.5%)	n.a.
Bacsalmas (Hungary)^F;G^	1600–1700 AD	12	18/480	46	12/30 (40.0%)	2/16 (12.5%)
Rain/Lech (Germany)^C^	1400–1800 AD	11	48/2.547	59	10/59 (17.0%)	n.a.
Sulzbürg (Germany)^H^	1550–1750 AD	0	0/25	25	0	3/25 (12.5%)

*: Typical TB cases and cases with macropathology that might represent mycobacterial disease. For the morphological characteristics of “typical” and “suspective” cases see the text. All data have been taken from own previous publications (some with recalculation in order to render data comparable) ([5]^A^, [15, 16]^B^, [17]^C^, [18]^D^, [22]^E^, [23]^F^, [4]^G^, [24]^H^).
